# Investigating partner involvement in pregnancy and identifying barriers and facilitators to participating as a couple in a digital healthy eating and physical activity intervention

**DOI:** 10.1186/s12884-021-03917-z

**Published:** 2021-06-28

**Authors:** Alexandra Rhodes, Andrea D. Smith, Clare H. Llewellyn, Helen Croker

**Affiliations:** 1 Research Department of Behavioural Science and Health, Institute of Epidemiology and Healthcare, University College London, Gower Street, WC1E 6BT London, UK; 2 Population, Policy and Practice Department, UCL Great Ormond Street Institute of Child Health, 30 Guilford Street, WC1N 1EH London, UK

## Abstract

**Background:**

Maternal obesity and excessive gestational weight gain are associated with adverse maternal and foetal health outcomes. Interventions targeting dietary and physical activity behaviours during pregnancy have typically been directed at women only. A digital intervention targeting couples could encourage expectant parents to support each other in improving energy balance (dietary and physical activity) behaviours.

**Aims:**

This study aimed to investigate the role partners play in pregnant women’s energy balance behaviours, and to identify barriers and facilitators to participating as a couple in a digital intervention to encourage healthy eating and physical activity in pregnancy.

**Methods:**

A qualitative design combined online focus groups and telephone interviews. Three focus groups were held with men (*n* = 15) and one mini focus group (*n =* 3) and 12 telephone interviews were conducted with women. Participants were either in the last trimester of pregnancy or had a baby under 18 months old. Most were from more deprived population groups where prevalence of maternal obesity is higher. Data were analysed thematically. Barriers and facilitators to participating as a couple in a digital intervention were mapped to the COM-B model and the Theoretical Domains Framework.

**Results:**

Four main themes were identified; partner involvement and support; partner understanding of good energy balance behaviours; couple concordance of energy balance behaviours; partner influence on her energy balance behaviours. Most facilitators to participating in a digital intervention as a couple fell within the Reflective Motivation domain of COM-B. Men were motivated by the desire to be supportive partners and good role models. Women were motivated by their belief that partner involvement would improve their success in achieving goals and enhance couple-bonding. Other facilitators included concordance in dietary behaviours (Physical Opportunity), healthcare practitioner recommendation, perceptions of pregnancy as ‘ours’ (Social Opportunity) and feeling supported and involved (Automatic Motivation). Barriers were rarely mentioned but included potential for partner conflict, perceptions of pregnancy as ‘hers’ and economic constraints.

**Conclusions:**

An opportunity exists to harness partner support to improve maternal energy balance behaviours. Barriers and facilitators to participating in a digital intervention as a couple indicate its potential to benefit emotional and relationship wellbeing in addition to physical health.

## Introduction

In the UK, around half of women begin pregnancy with overweight or obesity [1]. Obesity in pregnancy is linked to a range of adverse maternal and foetal health outcomes [2]. Moreover, starting pregnancy with overweight or obesity increases the risk of excessive gestational weight gain (GWG) [3], which, independent of pre-pregnancy weight, is associated with complications during pregnancy and birth such as gestational diabetes and Caesarean section [4], as well as life-long weight-related health issues for mother and baby [5].

Pregnancy is a time when many women are motivated to make positive lifestyle changes to optimise the health and well-being of their unborn child [6]. Accordingly, pregnancy has often been described as a ‘teachable moment’ [7]. However, most interventions encouraging healthy eating and physical activity in pregnancy tend to be concerned with their short term effect on GWG rather than the development of longer term healthy-lifestyle habits [8]. Whilst reducing excessive GWG is clearly an important goal, pregnancy also represents an opportunity for women to re-set dietary and physical activity behaviours as they embark on their new role as a parent.

Recent years have seen the growth of mhealth and the emergence of digital interventions targeting healthy eating, physical activity and weight management in pregnancy [9],[10]. Currently these do not appear to be as effective as face-to-face interventions. However, their advantages of lower cost and broader reach confirm the need to identify ways of rectifying this [11]. A novel approach to enhancing the effectiveness of digital interventions could be to enrol couples rather than pregnant women alone. Partner support in pregnancy has been shown to be associated with maternal and infant well-being [12],[13],[14]. However, studies investigating its effect on health behaviours have, to date, largely focused on smoking and alcohol [13],[15].To the authors’ knowledge, little is known about the effect of partner support on energy balance (dietary and physical activity) behaviours, or more broadly the role of partners in women’s dietary and exercise behaviours during pregnancy.

Hence, the aims of this research were twofold; firstly to provide insight into partners’ role in energy balance behaviours during pregnancy; and secondly to identify the barriers and facilitators to participating as a couple in a digital intervention targeting healthy eating and physical activity during pregnancy. We purposefully set out to explore the barriers and facilitators to couple-participation, as an extensive body of literature on barriers and facilitators to engaging in healthy eating and physical activity interventions in pregnancy and generally already exists [16–18].

## Methods

### Theoretical framework

This study used the COM-B model and the Theoretical Domains Framework (TDF) to investigate barriers and facilitators to participating as a couple in a healthy eating and physical activity intervention during pregnancy [19]. COM-B posits that behaviour (B) is part of an interacting system involving a person’s capability (C) to perform the behaviour, the opportunity (O) afforded to them to perform the behaviour and their motivation (M) to perform the behaviour. In order to change behaviour, one or more of these components needs to change. The TDF provides an additional layer of granularity to COM-B, tying it to 14 domains of key constructs from behaviour change theories [20].

### Study design

We used a mixed methods qualitative design combining focus groups and individual interviews. The study was undertaken between July and September 2020, hence COVID-19 restrictions necessitated a deviation from the original study design of face-to-face focus groups and interviews. Instead we conducted focus groups online using a video conference platform, and individual interviews by telephone.

We chose focus groups for men on the basis that they would create a more relaxed and interactive environment in which to explore views and experiences of a topic they were likely to be unfamiliar discussing. Whilst such interaction also brings with it the risk of peer influence, using an experienced moderator with an understanding of how to create an environment in which participants feel able to express their own views and how to mitigate posturing and peer influence, ensured that the benefits of a group approach outweighed potential negative effects. In contrast we chose one-to-one interviews for women, as we were keen to investigate their individual experiences of partner support both in general and with respect to energy balance behaviours.

We developed topic guides for the focus groups and telephone interviews, using open-ended questions to explore the experiences and role of partners in pregnancy, changes to lifestyle behaviours made by both men and women, sources of information about energy balance behaviours in pregnancy, including usage of pregnancy apps and response to the concept of an app-based intervention for couples to encourage the development of healthy eating and physical activity habits. The intervention concept was introduced to participants as *‘A new app feature to help expectant parents develop healthy eating and exercise habits, with information and tips and the ability to set yourselves goals and track your progress’.*

Questions around the barriers and facilitators to the couples intervention were guided by COM-B and TDF [21] The discussion flow was largely participant-led, with the moderator ensuring it stayed on topic and that all key topics were covered.

### Participants

To ensure that study participants had experienced a whole or close to whole term of pregnancy and that it was still fresh in their minds, we recruited expectant parents in the last trimester of pregnancy or parents of babies ≤ 18 months old. Since maternal obesity is more prevalent in more deprived population groups, we aimed to recruit the majority of participants from low income groups and/or without tertiary education and/or from minority ethnic groups [22]. Given the constraints of sample size and the importance of creating a safe space for participants to share their views, we restricted the sample to heterosexual male partners only at this stage of research. Same sex partners will be included in the next stage of the intervention development process and are represented in our PPI (public patient involvement) programme.

### Recruitment procedure

Due to restrictions as a result of COVID-19 an entirely remote recruitment methodology was implemented. Given the challenges of recruiting men from low income and minority ethnic groups into research, we made an a priori decision to use a professional market research agency to recruit the men’s focus groups. Participants were offered a £25 incentive. We recruited telephone interview participants from a convenience sample that had previously responded to a national online survey conducted by three organisations - Best Beginnings, Home-Start and Parent Infant Foundation. This online survey, conducted April to June 2020, explored the views and experiences of expectant parents and parents of very young babies during the COVID-19 pandemic [23]. At the end of the survey respondents indicated whether they would be willing to take part in a follow-up telephone interview for Best Beginnings. Those agreeing were emailed by Best Beginnings and asked for their consent to pass on contact details to the team at UCL who would be conducting telephone interviews for the present study. This generated a list of approximately 250 names from which our sample was drawn. Priority was given to those meeting one or more of the indicators of deprivation outlined above. Due to limited ethnic diversity within this convenience sample, we added an extra mini focus group of Asian women, recruited by the professional market research agency.

### Data collection

AR, an experienced qualitative researcher, moderated all of the focus groups and conducted the telephone interviews between July and September 2020. All sessions were audio recorded.

### Data analysis

We analysed the data using an inductive thematic approach [24]. All focus group and telephone interview audio recordings were transcribed and uploaded to NVivo 12 Pro [25]. After re-reading all the transcripts and trial coding 2 focus groups and 3 telephone interviews, AR developed an initial coding framework based on the responses of both men and women. Using this framework but refining it as necessary to accommodate new ideas emerging from the data, AR and AS independently coded 2 focus groups and 6 telephone interviews (i.e. 50 % of the data). Any discrepancies between AR and AS over codes or coding definitions were resolved through discussion. AR then coded the remaining 50 % of transcripts, including those trial-coded in the coding framework development phase. AR and AS identified the emerging themes through an iterative process of discussion, refinement and development. AR revisited the transcripts to confirm the legitimacy of the final themes. HC was involved at various stages throughout the coding and theme development process, to verify its accuracy and appropriateness. The initial mapping of the barriers and facilitators according to TDF domains and the COM-B model was conducted by AR. AS and HC contributed their views, and discrepancies were resolved through discussion.

## Results

We conducted three focus groups amongst men (*n* = 15), and 12 telephone interviews and one mini focus group (*n* = 3) amongst women (*n* = 15). The focus groups lasted between 45 and 91 min (averaging 72 min), and the telephone interviews between 22 and 43 min (averaging 32 min).

### Participant characteristics

Participant characteristics are summarised in Table 1. Men in the sample were aged 27–44 years (mean 35 years) and of varying ethnicities (5 were Black, 5 were White, 4 were Asian and one was mixed ethnicity). None of the men had post-school qualifications and 3 had no formal educational qualifications. All of the men were first time fathers/fathers-to-be. Nine of the men lived in London or the South East and 6 lived in the Midlands or North England.

Women in the sample were aged 18–39 years (mean 31 years), and ethnicity-wise, 9 were White, 3 Asian and 3 Black. Seven women had no post school qualifications and one declined to answer. Five women were multiparous. The women in the sample were geographically spread across the UK (excluding Northern Ireland).

**Table 1:** Participant characteristics

	Men	Women	Total (%)
**Age (years)**			
< 20	0	1	1 (3)
20–29	2	5	7 (23)
30–39	10	9	19 (63)
40+	3	0	3 (10)
**Ethnicity**			
White	5	9	14 (47)
Black	5	3	8 (27)
Asian	4	3	7 (23)
Mixed	1	0	1 (3)
**Education (highest qualifications)**			
No formal qualifications	3	1	4 (13)
GCSE	7	5	12 (40)
A level	5	1	6 (20)
Graduate	0	5	5 (17)
Post-graduate	0	2	2 (7)
Not stated	0	1	1 (3)
**Pregnancy/baby status**			
First pregnancy/baby	15	10	25 (83)
Second + pregnancy/baby	0	5	5 (17)
Last trimester	0	2	2 (7)
Baby < 6 months	7	6	13 (43)
Baby 6–12 months	3	7	10 (33)
Baby 13–18 months	5	0	5 (17)

### Themes

Four themes relating to partners’ role in energy balance behaviours in pregnancy were identified; partner involvement and support; partner understanding of good energy balance behaviours; concordance of energy balance behaviours in couples; partner influence on her energy balance behaviours. These themes arose from comments made by all participants in our sample, that is men talking about their own attitudes, experience and behaviours and women talking about their views and experiences of their partners’ attitudes and behaviour. No obvious differences emerged according to demographic variables or whether participants were first time parents and expectant parents and those who already had children, although the sample size of the latter group was very small (*n* = 5). The themes provide a context for understanding barriers and facilitators to participating as a couple in a healthy eating and physical activity intervention during pregnancy.

### Partner involvement and support

Experiences and attitudes of both men and women in our sample suggested a continuum of partner involvement and support in pregnancy, with three broad typologies of expectant fathers emerging:

Our baby, her pregnancy: The least involved expectant fathers were characterised by their view of the pregnancy being their partner’s. They perceived pregnancy to be a ‘woman’s thing’ and often talked about the active involvement of other female relatives in their partner’s pregnancy. As they saw it, their role as fathers began only when their baby was born. For some participants these gender-stereotypical roles were rooted in family or cultural beliefs.

*When we visited my in-laws, there was lots of questions …. mostly aimed at my wife….I did feel that I was on the outskirts, I wasn’t shocked, as such…. I’d be sitting there watching football with the beers. (Male,#2)*

Whilst they were happy to support their partner in any way she wanted, they tended to adopt a reactive approach, taking the lead from her. Similarly whilst they were receptive to information, advice and guidance from their partner, other family members and healthcare practitioners (HCPs), they rarely sought it themselves.

*I think it’s widely known in the medical world and even outside of the medical world, that the expectation for self-educating yourself in pregnancy is the woman’s role. (Male, #10)*

Women whose partners conformed to this typology tended to criticise the men for not understanding pregnancy and not being more proactive in supporting them.

*They don’t understand anything, the sleeping problems the sitting problems. Because my husband doesn’t understand, I do see it more of my pregnancy but our baby if that makes sense. (Female, #15)*

Our pregnancy, her body: Sitting midway along a dimension of involvement, another group of participants expressed a view that whilst they felt pregnancy was a shared event, the physicality of the experience belonged to the woman. Expectant fathers in this group recognised their important role in providing practical and emotional support to their partner, but ultimately recognised that the physical experience of pregnancy belonged only to their partner. For some this created a dissatisfying sense of being a side-player or worse, excluded.

*I see it my role… is like a supportive role, like an auxiliary role, where you can’t obviously take the main role, so you basically work like a mix between a waiter … and being someone who’s there for her. (Male, #6)*

*Sometimes men can feel a bit left out of the whole pregnancy because the woman goes through all the changes and has these massive mood swings…I felt like a bit of a spare part to be honest. (Male, #13)*

For this group and for the previous group, men’s experience of pregnancy support services and HCPs could reinforce their views on their role in pregnancy.

*I felt that it (NCT) was that was very much geared towards the woman and in fact, I made the point that is there any point of me being here? (Male, #10)*

Our pregnancy: The most involved expectant fathers in our sample were characterised by their belief that their role as fathers had already begun during pregnancy.

*When we’re talking about the baby he’s always talking about our son, he’s very much going to be part of the child’s life, he wants to be part of it now. He talks to the bump. (Female, #5)*

As such they were fully involved in all aspects of the pregnancy and made sure that HCPs understood and respected this. In addition to providing practical and emotional support to their partners, these expectant fathers took steps to inform and educate themselves about pregnancy. Some had downloaded pregnancy apps or joined social media groups for expectant dads.

*It was definitely a team effort. Guys in the 21st century are a lot more on it. You look at the apps and you help where you can. (Male, #7)*

*I made myself be involved – I wanted to know everything that was going on. So I wasn’t going to wait - I was in first, asking them* (HCPs) *everything (Male, #14)*.

Most women in our sample whose partners conformed to this typology supported their partners’ view of seeing pregnancy as a shared adventure. They welcomed the level of support they received and were often aware of their partner’s need for support too.

*It was our pregnancy - it was our baby. We were pregnant – not just me. (Female, #7)*

*He’s very hands on. He’s come to all my* (antenatal) *appointments … As we’ve had more children he has looked after them so I can go and do something …it’s divide and conquer…very involved in every aspect. (Female, #12)*

However, one participant voiced a concern about her partner’s involvement bordering on controlling or ‘policing’ behaviour.

*It’s a little bit like he was policing me, but then again it goes back to this is our pregnancy, not mine - it was because he cared for me and baby. (Female, #7)*

### Partner understanding of good energy balance behaviours

Levels of understanding regarding the importance of good energy balance behaviours in pregnancy, that is to eat a healthy, balanced diet and remain physically active (National Institute for Healthcare and Excellence guidelines [26]), varied in both men and women. Apart from the most involved expectant fathers, most relied on their pregnant partner as their main source of information on this matter. Most men were aware of the relationship between maternal diet and baby’s health and development and talked about ‘healthy eating’. However few understood the risks to maternal and foetal health of poor energy balance behaviours. Awareness of conditions like gestational diabetes was largely limited to those who had experience of it. Likewise, partners’ knowledge of what is a healthy diet in pregnancy varied considerably. More health conscious men and those who were more involved in the pregnancy talked about the value of specific food groups (typically fruit and vegetables) and nutrients. Others were more vague, often referring to a ‘normal’ or balanced diet or simply highlighting dietary changes she had made.

*I think we just kept it as normal cos we was (sic.) already eating quite nutritious diet…as long as they’re having these Pregnacare tablets, then that covers most of it. (Male, #6)*

*She had a craving for double cheeseburgers from Mac Donald’s, but she was making sure she was taking Pregnacare and Folic acid. (Male, #9)*

Understanding of the importance of exercise for maternal and foetal health was limited. Typically only those who already were quite committed to exercise knew of its benefits in pregnancy. Others were unsure what type or amount of exercise was safe in pregnancy, tending to believe it was safer to avoid exercise apart from walking.

*It’s taken us, it took us a long time to get to this point and we didn’t want to throw it away for the sake of doing you know, one gym session a week, or running or, or even fast walking. (Male, #10)*

*I see some pregnant women doing push ups and running and I stand there in amazement thinking is it really worth taking that risk? (Male, #7)*

### Concordance of energy balance behaviours in couples

The majority of participants reported a high degree of concordance of dietary behaviours with their partner, and since many women had made changes to their diet during pregnancy, many expectant fathers had, by default, done so too. Generally these had been healthier changes, such as increasing fruit and vegetable intake or decreasing snack foods and takeaways. However participants also reported worsening dietary behaviours.

*So whatever made her sick is basically what we what we stopped eating. (Male, #1)*

*I think my wife’s diet sort of lapsed or relaxed….I think that why a lot of guys like me put on weight during pregnancy because my wife’s lifestyle choices meant she didn’t have the energy to cook and therefore it was easy for me to pick up the phone and ring for a pizza or whatever. (Male, #10)*

*He put on a bit of sympathy weight during pregnancies…. I was getting hungry and I think he just started to eat more as well….bigger portions at mealtimes. (Female, #10)*

For the majority, concordance in dietary behaviours was driven by convenience of preparing one meal rather than a view that expectant fathers should also make dietary changes. Indeed men generally did not feel the need to sacrifice their own preferences.

*She tried to eat a lot more healthy, like vegetables which she wouldn’t normally do. I tried but I must admit I don’t like salads and that. (Male, #11)*

However, for a small minority of more involved expectant fathers, concordance was motivated by a desire to show support and solidarity.

*I mean, it’s definitely difficult when you will want to get the take-away or something but she can’t really eat it. Sometimes you’d have to sacrifice your pleasure. (Male, #4)*

*Well he actually started to take smoothies as well, and the disgusting vitamins because like, he didn’t want me to feel singled out. (Female, #2)*

Unlike in dietary behaviours, participants rarely reported couple-concordance of physical activity behaviours, either in the type or amount of exercise taken or general activity levels. The only exceptions to this was firstly the tendency for men to share more household duties and secondly couples taking walks together, particularly towards the end of the pregnancy.

*….allowing her to not do the things she might have done before, that are a bit strenuous. So kind of taking work away from them. (Male, #6)*

*Hold her hand and walk with her to the park …because the doctors and the nurses say she has to walk a bit – 100 to 200 m a day - because she was suffering from diabetes. (Male, #12)*

### Partner influence on her energy balance behaviours

Only a small number of more health conscious men in our sample actively encouraged their pregnant partners to adopt better energy balance behaviours.

*When I was pregnant, he was then cooking more because I was either having a nap or I was tired or whatever. So he would be consciously trying to put veg and stuff like that into my diet. (Female, #3)*

Diet-wise, the majority of men felt their role was to concur with her choices. This was particularly apparent in men’s tendency to enable their partner’s cravings.

*That’s the best way to placate them – if they say KFC you just go get KFC. If they say Burger King, you just go get it – no opposition no question just get it. (Male, #14)*

Indeed some men reported that even when they felt their partner was eating the wrong food or gaining too much weight, they would keep quiet to keep peace.

*I’ll tell you what anyone who says they are not going to say let it slide for the day…I’d rather fight the battles I can win. If they are already feeling body conscious, they already know a packet of custard creams isn’t going to do them any good, there’s not point me telling them that and making them feel worse. (Male, #13)*

There was evidence of some men unintentionally encouraging unhealthy dietary behaviours, which appeared to be rooted in cultural beliefs.

*Everyone kept telling me to eat for two – maybe it’s in our culture - Bangladeshi culture. (Female, #15)*

*My mother-in-law, even my husband, all my family were saying you’ve got to eat for two. (Female, #13)*

Similarly some men were encouraging their partners to avoid exercise or any overly strenuous activity, partly because they felt allowing her to relax and rest was evidence of their support and partly because their belief that it was unsafe in pregnancy.

*I don’t know what other exercises are acceptable in pregnancy, I have no idea, so we stuck with walking. (Male, #1)*

### Barriers and facilitators to participating as a couple in an intervention mapped to COM-B and TDF

Table 2 summarises the identified barriers and facilitators to participating as a couple in a digital intervention to encourage healthy eating and physical activity in pregnancy. Barriers and facilitators were largely different for men and women, although some were common to both. The most highly populated COM-B domain was Reflective Motivation, with TDF Belief about the Consequences being particularly pertinent to women and Identity, Goals and Intentions to men.

**Table 2:** COM-B and TDF mapping of barriers and facilitators to participating as a couple in a digital healthy eating and physical activity intervention

COM-B	TDF Domain	Barriers	Facilitators
Motivation Reflective	Identity/goals Beliefs about consequences Intentions	‘Her pregnancy’ -fatherhood begins at baby’s birth (Partner) Potential conflict/controlling behaviours (Woman)	‘Our pregnancy’ (Both) Being a fit and healthy father (Partner) Being a supportive partner (Partner) Being a good role model to child (Both) Increase commitment and hence improve success rates (Woman) Give partner (more of ) a role in pregnancy (Both) Positively impact relationship (bonding) (Both) Improve partner knowledge and hence support (Woman) Commitment to be a supportive partner during pregnancy (Partner)
Motivation Automatic	Emotions		Feeling supported (Woman) Feeling included (Partner)
Opportunity Social	Social influences Social norms	‘Her pregnancy’ and cultural/family gender stereotyping (Both)	HCP recommended (Both) ‘Our pregnancy’ – partners today are involved (Both)
Opportunity Physical	Context and resources	Cost and time of participating together (Both)	Existing concordance in energy balance behaviours (Both)
Capability Psychological	Knowledge		Understand the importance of good energy balance behaviours to mother’s and baby’s health (Partner) Understand the importance of partner support in her achieving healthy energy balance behaviours (Partner)
Capability Physical			

### Reflective motivation

#### Identity, goals and intention

A key motivation for men to participate in the intervention was their desire both to be a supportive partner and to have a role within the pregnancy. Whilst some regarded participating in the intervention to be evidence of their existing commitment to their partner and their pregnancy, others saw it as a way of increasing their involvement and support. Women too recognised their partner’s intention to be supportive as a facilitator to his participation in the intervention.

*To have something that is partner oriented for the two of you, that would really help. A lot of guys want to be involved now, it would appeal to so many people. (Male, #9)*

*He would definitely cut down on KFCs if he thought it was better for me and the baby … anything to make it work and support me with this. (Female, #4)*

The opposite of this was the belief, alluded to rather than explicitly identified as a barrier to participation, that a man’s role as a father does not begin until the baby is born.

*At my early stages I didn’t get much attention but now he can see the bump I get more attention*. *(Female, #14)*

*At risk of sounding uncaring, I didn’t really have to change much to be honest….I think she more or less had it under control. I didn’t really have to do anything. (Male, #1)*

A desire to be a fit and healthy parent and a good role model for their child was a facilitator, for men especially. Even those who subscribed to the ‘her pregnancy’ point of view could be motivated by the potential benefits to themselves of jointly engaging in a healthy eating and physical activity intervention.

*I don’t want to be a fat unhealthy dad…I want to be healthy into my old age. (Male, #3)*

*I’m not gonna lie – come that sports day when they go to primary school, I don’t want to be the last dad! (Male, #10)*

*You don’t want to be a lazy dad sitting on the sofa (Male, #11)*

#### Beliefs and consequences

Particularly motivating to women was a belief that working together as a team would increase the likelihood of success of adhering to the chosen health behaviour.

*I think it’s a good idea because mutual support is the best way to make changes. It’s almost impossible to make a change to your diet if one of you is doing it and one of you isn’t. (Female, #5)*

Some women recognised their need to be accountable to someone else in order to stick to commitments.

*As soon as I have told somebody it’s like I’ve committed to it, but if I’ve only kept that to myself Then I can say to myself well I didn’t really want to do it or I’ll find an excuse not to. (Female, #6)*

Women also recognised the benefit to their partners of participating jointly in that this would create a tangible role for partners and help them to feel involved.

*Because partners I think, like, males are the other partner that isn’t pregnant, like some of them don’t feel like very included but if they had the app as well, they would probably feel more involved as well. (Female, #2)*

More broadly, both men and women also identified the potentially beneficial consequences on the partner relationship of working towards goals together. The opportunity to bond through a shared goal was particularly appealing both to those who found pregnancy a bonding experience and those who had struggled more with the changes it had brought to their relationship.

*I think it’s a good idea – you can both get involved and it would bring you both together (Male, #9)*.

*And I think people would respond well to having something to do together because there is that feeling that they are bonded together at a time when other things are changing and it can be quite scary. (Female, #6)*

Women whose partners were at the least involved end of the spectrum were motivated by the thought that a couple’s intervention could improve their partner’s understanding about pregnancy and thereby lead to him supporting her better.

*I think the app will help for the husbands because they will understand more and this way we’d get more support from them, because they’d understand more. (Female, #13)*

*If someone was to tell him maybe don’t have too much sugar during your pregnancy he might be like you’re not actually meant to have this, give it to me. Because they don’t know all they hear from their mothers is eat for two, eat for two and they probably think they are doing the right thing by feeding me. (Female, #15)*

Some men also recognised the opportunity the intervention might offer for self-education around pregnancy and improvement of their skill set.

*All the advice that we can get is better because, you know, we can’t be expected to know everything. (Male, #6)*

The only barrier identified in the context of beliefs about consequences was an anticipation that participation might lead to conflict between couples, particularly if the intervention focussed on weight gain issues.

*You would have to have good faith on your partner that they’re able to motivate you without it being of a matter of them nagging you - you’re putting on weight, dear and you need to be healthier….It would start off nicely and he’d be encouraging and eventually he would grate on me and I’d be saying I’m not doing it. (Female, #11)*

One woman was concerned that the intervention might give her partner permission to be overly controlling over her dietary and exercise behaviours.

### Automatic motivation

#### Emotions

Here, women identified the warm feelings of togetherness and being supported as a facilitator whilst men talked about being included.

*I need your support and I want you to do it as well, so I don’t feel like I’m on my own. (Female, #8)*

*I’d say you’d feel a lot more involved…. my own experience, I felt like I didn’t really have any role. (Male, #1)*

*You need to feel that you and your partner are close together and you are not alone in anything. (Female, #6)*

### Physical opportunity

#### Context and resources

Couples’ concordance of dietary behaviours and in taking walks was perceived to be a facilitator in a practical sense of fitting in with existing behaviour and lifestyle patterns.

*We cook together we eat together so we’re eating the same, the same meals. (Female, #5)*

However, the time involved in coordinating activities and the cost of both eating healthier and potentially more expensive foods were occasionally cited as barriers.

*I think that the barriers will be maybe time and trying to get organised, (Female, #1)*

*(He) would have his own rice… white rice and I’d have a wholemeal rice…. When you’re looking after the pennies cos you’ve got a baby on the way it* (wholemeal) *can be quite an expensive option. (Female, #8)*

### Social opportunity

#### Social norms

Perceiving pregnancy as ‘ours’, a shared experience, emerged as a facilitator to participating as a couple. Conversely, perceiving pregnancy as ‘hers’, a view that appeared to be propagated by family and cultural gender stereotyping, might be a potential barrier, although this was alluded to rather than openly voiced in our research.

#### Social influences

HCP endorsement was identified as an important facilitator to participating as a couple. Men in particular claimed that they would be even more likely to participate in the intervention if it was suggested to them by the midwife or GP. Not only would this validate the credibility of the intervention, but also it would confirm the importance of the partner’s role in pregnancy. Similarly women could feel that HCP recommendation would legitimise their desire for greater involvement and support from their partner.

*It would be like a stamp of approval from the NHS. When it’s being recommended by a health professional it carries a lot of weight. (Male, #8)*

*If it was for the father, coming from the midwife makes us be part of the process I would value that because I am being included. (Male, #3)*

### Psychological capability

#### Knowledge

Having good understanding of the importance of good energy balance behaviours to maternal and foetal health appeared to be a facilitator for men in that it enabled them to appreciate value of working with their pregnant partner to reduce the risk of adverse health outcomes. Understanding of the potential impact of partner support during pregnancy worked similarly as a facilitator.

*It should talk about how it helps your baby and how it helps your relationship rather than just it’s good for you. You hear that message – it’s good for you – all the time. (Male, #13)*

### Physical capability

Participants did not identify any barriers or facilitators to participating in the intervention as a couple that fell within the physical capability domain.

## Discussion

This study explored partners’ role in energy balance behaviours during pregnancy and, using COM-B and TDF, identified barriers and facilitators to participating as a couple in a digital intervention targeting healthy eating and physical activity. We identified four themes around partners’ role in energy balance behaviours; partner involvement and support; partner knowledge of the importance of good energy balance behaviours; concordance of energy balance behaviours in couples; partner influence on her energy balance behaviours. Differing levels of partner involvement and support during pregnancy broadly correlated to partners’ level of understanding of the importance of energy balance behaviours to maternal health. Concordance of dietary behaviours was widely reported, although couples’ exercise behaviours tended to be less alike. Partners rarely seemed to be a positive influence on women’s dietary choices, typically deferring to her desires, particularly with regard to cravings. Caution over the safety of exercise in pregnancy meant men could be a negative influence on women’s exercise behaviours. Most facilitators to participate as a couple in an intervention lay within the Reflective Motivation domain of COM-B. Men’s motivations derived from their desire to be involved in the pregnancy as a supportive partner, and to be a fit and healthy father who is a good role model for his child (TDF: Identity, Goals and Intentions). Women were motivated by the belief that a couples intervention might increase their partner’s understanding and support, and a joint effort might improve their likelihood of success in achieving energy balance goals (TDF: Belief in Consequences). The opportunity for greater couple bonding was also highlighted as a potential motivator, although couple conflict was a barrier for women who anticipated their partner being controlling. Within the Social Opportunity domain, HCP recommendation was seen as a key facilitator, both legitimising the role of partners in the pregnancy and adding integrity to the intervention. Conversely, cultural beliefs around the role of men in pregnancy were identified as potential barrier (TDF: Social Influences). Within the Psychological Capability domain understanding the importance of good energy balance behaviours to maternal health and the value of partner support was a facilitator to intervention participation (TDF: Knowledge).

Our research echoes the findings of previous studies reporting men’s desire to be involved in pregnancy and to support their partner [27],[28]. Men in our sample who were not at the most involved end of the spectrum believed that participating in an intervention with their partner would give them a greater role within the pregnancy and help them feel more valued. A meta-synthesis of expectant fathers’ experiences of pregnancy concluded that if HCPs made greater efforts to involve fathers, this would increase their sense of being valued and place them in a better position to support their pregnant partners [29]. Our research identified HCPs’ recommendation as a key facilitator to participating in a couples intervention in that it both acknowledged the importance of partners in pregnancy and endorsed the trustworthiness of the intervention. Men also identified more self-centred motivations to participating in the intervention – a desire to be a fit and healthy father who is a good role model for their child. Given that weight gain in men during pregnancy is quite common, the potentially positive effects of a couples intervention on men’s energy balance behaviours is also important [30].

A key finding of our study is that women believe they would be more likely to succeed in improving their energy balance behaviours during pregnancy if their partner was working with them towards shared or similar goals. Few studies have examined the effect of partner support on dietary and/or physical activity behaviours in pregnancy, although two interventions that included partners both showed promising results. In Bangladesh engaging husbands in a maternal nutrition program substantially contributed to their partners’ better dietary practices during pregnancy [31]. The authors showed that husband’s support, as a consequence of their knowledge, awareness, self-efficacy and social norms, explained nearly half of the program impact on maternal supplement intake and a quarter for diet diversity. Smarter Pregnancy, an mhealth intervention in the Netherlands targeting vegetable and fruit in pregnancy reported greater effect amongst couples than women undertaking the intervention alone [32]. Studies in preconception couples and non-pregnant populations have also shown the positive effect of couple-inclusion on energy balance behaviours [33],[34] and a randomised controlled trial of a mobile preconception lifestyle programme for couples is currently underway in Belgium [35].

Beyond the physical benefits of participating in an intervention as a couple, our research indicated there may be benefits to couples’ emotional and relationship well-being. The association between partner support and maternal mental wellbeing has been demonstrated in previous research [14]. A recent study investigating the effect of a couples intervention on partners of people with type 2 diabetes reported significant improvements in relationship satisfaction and lower depressive symptoms amongst those who had participated as couples rather than individuals [36]. The potential benefits of greater partner involvement in the pregnancy and couple bonding should not be underestimated.

Our research concurred with findings from previous studies that women’s dietary behaviours in pregnancy are influenced by their partners, friends and family [18], [37]. Hill suggests a new socioecological framework for maternal obesity which recognises the layers of influences on women’s behaviour [38]. An intervention aimed at couples could begin to shift the focus from women only and recognise these broader influences. Hill argues that a shift in focus will help to reduce weight stigma and blame, issues that our sample identified as a source of couple conflict and as such a potential barrier to the intervention. Shifting the emphasis from GWG and maternal responsibility to improving energy balance behaviours for couples or parents as they embark on this new life stage is more likely to benefit long term family health and contribute to the fight against childhood obesity.

Our research has important implications for the type of messages that may promote couple-uptake of a digital intervention to encourage healthy eating and physical activity. By mapping barriers and facilitators to participating as a couple, we have shown the importance of the reflective motivation domain as a source of both potential motivators and barriers to engagement.

## Strengths and limitations

A strength of this study is that it makes a significant contribution to the sparse body of literature investigating partners’ role in energy balance behaviours during pregnancy. A further strength of this study is the use of COM-B and TDF to systematically examine the barriers and facilitators. Moreover, our sample represents population groups who are most at risk of poor energy balance behaviours, excessive GWG and maternal obesity. It should be noted however that participants were pre-informed as to the discussion topics and as such there is risk of self-selection bias in our sample. Whilst our participants expressed a range of opinions and behaviours with respect to diet and exercise in pregnancy, we cannot be sure that on balance they were more inclined to positive energy balance behaviours than the general population. A further limitation of our sample was that all participants appeared to be cohabiting and in stable, loving relationships where the pregnancy had been welcomed. Consequently, even participants who represented the lower end of our spectrum of involvement in pregnancy appeared to be committed to their partners and the pregnancy. A limitation of any qualitative study is researcher subjectivity in the interpretation of the findings. To mitigate the effects of this we ensured our team of three researchers immersed themselves in the raw data and debated and challenged each other’s viewpoints as part of the analysis process. Finally whilst the ethnic diversity of our sample provided insight into cultural differences, our small sample size meant some minority groups were not represented. This will be addressed in later stages of our intervention design process.

## Conclusions

Our research suggests an opportunity exists to harness partner support to improve maternal energy balance behaviours. Facilitators to participating as a couple in a digital healthy eating and physical activity intervention indicate that its benefit could extend beyond physical health, to couples’ emotional and relationship well-being. Men appreciate the opportunity it would give them to feel more involved in the pregnancy and supportive to their partners. Women also value its potential to enhance partner involvement and support. In addition women are motivated by the prospect of a team effort improving their chance of success in achieving dietary and/or physical activity goals. Whilst this research has provided direction on the messages likely to motivate participation in a couples’ intervention, further research will be needed to determine how to optimise the appeal and usability of the intervention to ensure high and sustained levels of engagement.

## Data Availability

The data that support the findings of this study are available on request from the corresponding author.

## Abbreviations

UK: United Kingdom
GWG: Gestational weight gain
TDF: Theoretical Domains Framework
HCPs: Healthcare practitioners
